# Predicting functional and regulatory divergence of a drug resistance transporter gene in the human malaria parasite

**DOI:** 10.1186/s12864-015-1261-6

**Published:** 2015-02-22

**Authors:** Geoffrey H Siwo, Asako Tan, Katrina A Button-Simons, Upeka Samarakoon, Lisa A Checkley, Richard S Pinapati, Michael T Ferdig

**Affiliations:** Department of Biological Sciences, Eck Institute for Global Health, University of Notre Dame, Notre Dame, IN USA; Geisel School of Medicine, Dartmouth College, Hanover, NH USA; Epicentre, Madison, WI USA; Harvard Medical School, Boston, MA USA

**Keywords:** Plasmodium falciparum, Chloroquine resistance, *pfcrt*, Gene co-expression networks, Phenotypic states, Functional interactions, Regulatory interactions, Rewiring of gene networks

## Abstract

**Background:**

The paradigm of resistance evolution to chemotherapeutic agents is that a key coding mutation in a specific gene drives resistance to a particular drug. In the case of resistance to the anti-malarial drug chloroquine (CQ), a specific mutation in the transporter *pfcrt* is associated with resistance. Here, we apply a series of analytical steps to gene expression data from our lab and leverage 3 independent datasets to identify *pfcrt*-interacting genes. Resulting networks provide insights into *pfcrt*’s biological functions and regulation, as well as the divergent phenotypic effects of its allelic variants in different genetic backgrounds.

**Results:**

To identify *pfcrt*-interacting genes, we analyze *pfcrt* co-expression networks in 2 phenotypic states - CQ-resistant (CQR) and CQ-sensitive (CQS) recombinant progeny clones - using a computational approach that prioritizes gene interactions into functional and regulatory relationships. For both phenotypic states, *pfcrt* co-expressed gene sets are associated with hemoglobin metabolism, consistent with CQ’s expected mode of action. To predict the drivers of co-expression divergence, we integrate topological relationships in the co-expression networks with available high confidence protein-protein interaction data. This analysis identifies 3 transcriptional regulators from the ApiAP2 family and histone acetylation as potential mediators of these divergences. We validate the predicted divergences in DNA mismatch repair and histone acetylation by measuring the effects of small molecule inhibitors in recombinant progeny clones combined with quantitative trait locus (QTL) mapping.

**Conclusions:**

This work demonstrates the utility of differential co-expression viewed in a network framework to uncover functional and regulatory divergence in phenotypically distinct parasites. *pfcrt*-associated co-expression in the CQ resistant progeny highlights CQR-specific gene relationships and possible targeted intervention strategies. The approaches outlined here can be readily generalized to other parasite populations and drug resistances.

**Electronic supplementary material:**

The online version of this article (doi:10.1186/s12864-015-1261-6) contains supplementary material, which is available to authorized users.

## Background

Drug resistance often results from a mutation in a specific gene(s), such as a drug target or a drug transporter. The emergence of a resistance-conferring mutation within an evolutionary tuned system of functional and physical interactions can present enormous physiological challenges and can have broad phenotypic effects. For example, mutations in a single drug resistance gene can impact its interactions with other genes, physically and/or functionally, and can in turn constrain the evolution of highly connected genes [1]. Such interactions can affect the penetrance, expressivity and pleiotropic phenotypic effects of a single mutation [2]. Moreover, the spread of resistance alleles in populations creates novel interactions with accompanying fitness implications, as mutations recombine in new genetic backgrounds, sometimes decoupling co-evolved gene relationships. In some cases, recombination brings together beneficial alleles into one genome while in others, suboptimal combinations result [3]. The successful propagation of mutations from small founder populations implies that cells have mechanisms to buffer the impact of single gene mutations on their interaction partners. Notably, even when phenotypes such as drug resistance are controlled by a single major gene, individuals carrying the same genotype at that locus commonly present a range of phenotype levels [2,4-6].

Chloroquine (CQ) resistance is a prime example of the complexity of the evolutionary impact of mutations in a single drug resistance gene. All CQ-resistant (CQR) parasites reported to date contain a lysine to threonine substitution (K76T) in the *P. falciparum* chloroquine resistance transporter, *pfcrt* [7,8]. In spite of the high penetrance of this mutation, CQR parasites exhibit a wide range of resistance levels, indicating the involvement of additional genes [9]. Furthermore, the lone example of selection of CQ resistance in the laboratory was highly dependent on the genetic background that was drug pressured [9]. Unfortunately, in more than a decade since the association between *pfcrt* and CQ resistance was discovered [8,10,11], information about its extended functions, regulation and impact on other phenotypes or drug resistance evolution remain largely unknown. An understanding of *pfcrt’s* interaction partners could reveal genetic modifiers of CQ resistance and potential pleiotropic effects of the mutation.

The plasticity of gene regulation networks makes them powerful readouts of genome-wide responses to perturbations; moreover, global gene expression measurement is relatively simple, highly quantitative and unbiased view of regulatory outputs.

Here, we leverage gene expression data from CQR and CQ-sensitive (CQS) recombinant progeny clones to gain deeper insight into the biology of the *pfcrt* gene. We extend our work on genome-wide transcriptional profiling that found heritable regulatory variation controlling the expression of nearly 18% of the transcriptome [12]. The genetic locus encoding *pfcrt* emerged as a regulatory hotspot, suggesting that the associated transcriptional networks can provide more insights into its natural function and role in CQ resistance [12]. We leverage *pfcrt*’s co-expression relationships to examine its functional interactions in CQR vs CQS parasites and to predict regulatory and phenotypic divergence across the phenotypically distinct recombinant progeny clones.

## Results

**Figure 1:**
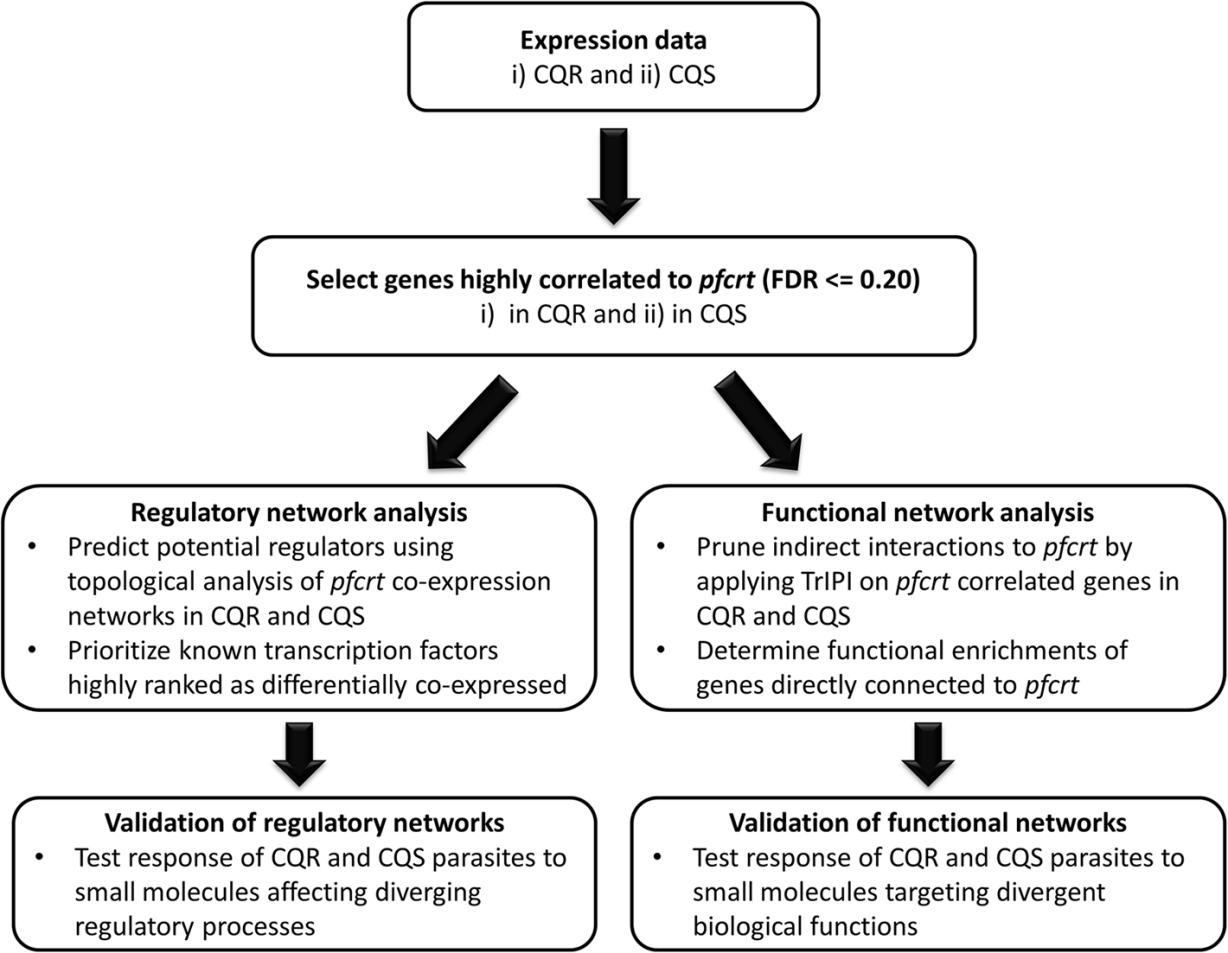
**Overview of the approach applied in this study to predict diverging functional and regulatory interactions associated with the drug resistance transporter gene,** ***pfcrt*** **.** Genes significantly correlated to *pfcrt* (FDR ≤ 0.20) in CQR or CQS recombinant clones were obtained using transcriptional data from the 24 hr developmental stage (trophozoites) [12]. Regulatory candidates associated with the *pfcrt* co-expression network in CQR or CQS parasites and functional partners of the gene were then predicted by applying the triangle inequality [22] based approach (TrIPI) developed in this study to assess the topological position of *pfcrt* correlated genes. Validations of the predicted regulatory and functional biological processes associated with *pfcrt* in CQR or CQS were then performed by measuring dose responses to small molecules targeting the processes. Additional information is provided in Additional file 1: section A.

In this study, we determine and compare gene sets that are co-expressed with the drug resistance transporter gene, *pfcrt,* in CQR vs CQS malaria parasites (Figure 1 and Additional file 1: section A) for three key reasons: i) genes showing similar patterns of co-expression often are functionally related [13,14] such that *pfcrt*’s co-expressed genes could highlight its endogenous roles and functions beyond its role in resistance; ii) differentially co-expressed genes could lead to divergent drug response and other phenotypes[14-17]; iii) because differential co-expression results from divergent regulation [18,19], the topological relationships between differentially co-expressed gene sets can reveal regulatory mediators of phenotypes [20,21]. Differential gene expression, the usual method for analyzing transcriptional data, cannot effectively address these questions because that approach considers genes individually rather than in context of their relationships [18,20,21]. Recently, gene co-expression networks have been used to distil information from transcriptional data to identify functional and regulatory interactions between genes, even when the key genes are not differentially expressed [20]. Their usefulness notwithstanding, co-expression networks are prone to a high rate of false positive correlations arising from indirect relationships between genes, requiring that we establish rigorous thresholds and a layered (sequential) method (summary in Figure 1 and additional details in Additional file 1: sections A, C and D) [22]. Specifically, to overcome false positive correlations, we develop an approach - Triangle Inequality Prioritization of Interactions (TrIPI) - that, given a correlation matrix of genes significantly correlated to a gene of interest (correlation coefficient, |*r*| ≥ 0.5, FDR ≤ 0.20), prioritizes sets of correlated genes into ‘direct’ functional partners of the gene and potential regulators of its co-expression network (Methods, Additional file 1: sections C and D). This method has 3 key advantages: i) it takes advantage of prior biological information by focusing on a gene of interest (here, *pfcrt*) to reduce the dimensionality of the hypothesis search space for significant gene correlations (Additional file 1: section C), ii) it prioritizes correlations between the gene of interest and other genes by examining the strength of the correlation in the context of how the two genes correlate to other genes, paring out indirect associations and thereby reducing false positive associations (Additional file 1: section C), and iii) it interrogates the topological relationships to identify candidate regulatory factors which are gene(s) that correlate significantly to a high fraction of the direct neighbors of the gene of interest (Additional file 1: section D). A comprehensive overview of the approach is provided in the Additional file 1: sections A, C and D. We apply this approach to predict candidate functional partners of *pfcrt*, drivers of its co-expression network and to provide molecular insights for phenotypic divergence between CQR and CQS parasite clones.

### *pfcrt* co-expression networks in CQR and CQS recombinants

**Figure 2:**
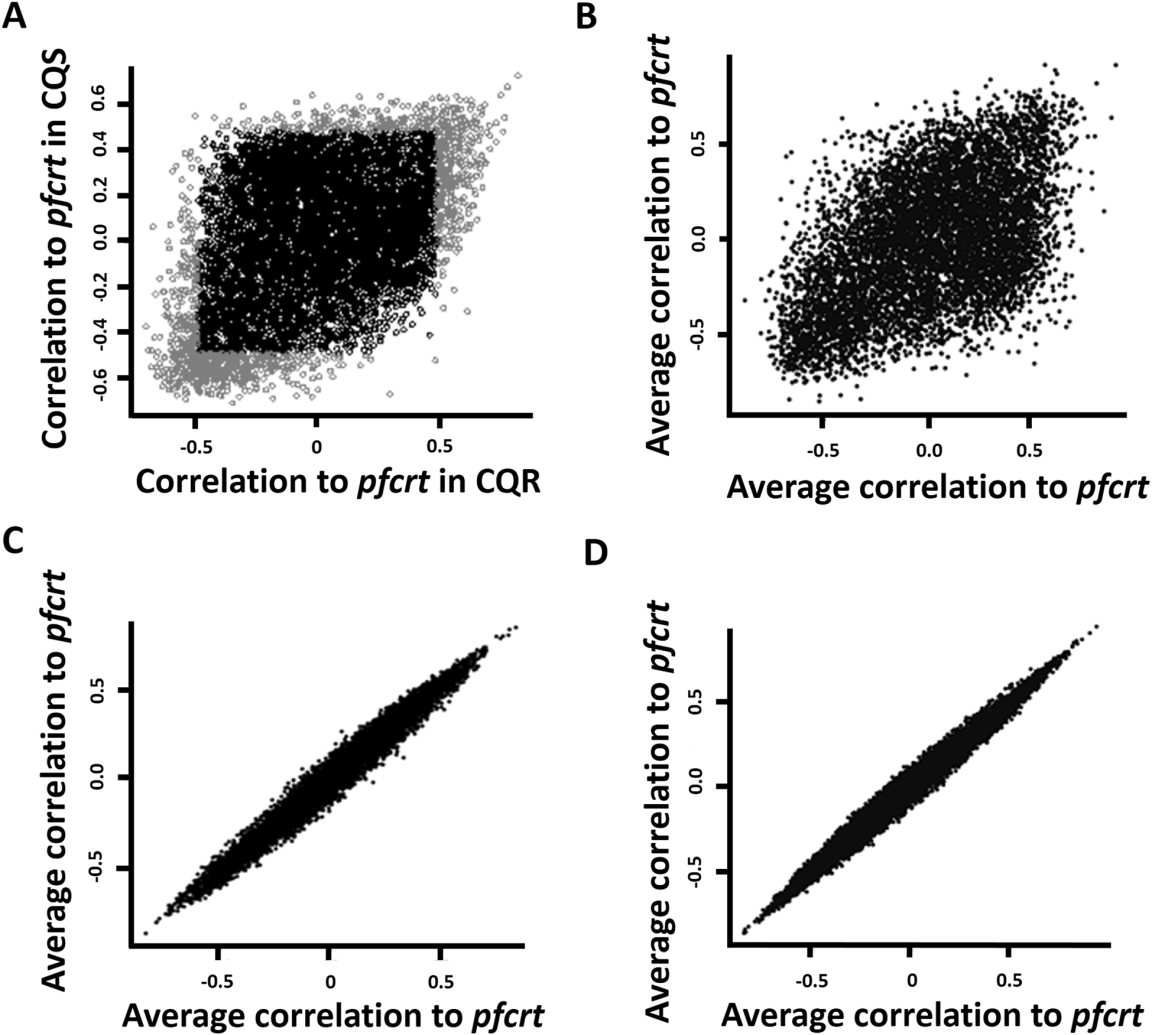
**Co-expression of all genes with** ***pfcrt*** **gene in CQR and CQS parasites. (A)** Correlation between the levels of each transcript in the genome to that of *pfcrt,* determined separately for CQS (x-axis) and CQR (y-axis) parasites. Grey region indicates genes whose correlation to *pfcrt* passed the threshold of FDR ≤ 0.20. **(B)** Average correlation between *pfcrt* and each transcript in 100 pairs of randomly sampled subsets of CQS (x-axis) and CQR (y-axis) parasites. Each subset of CQR or CQS parasites consists of transcriptional data from 8 parasite clones. **(C)** Average correlation between the transcript level of each gene to that of *pfcrt* in 100 pairs of randomly sampled subsets of CQS parasites. **(D)** Comparison of average *pfcrt* correlations in 100 pairs of randomly sampled subsets of CQR parasites. Like in **(B)**, each randomly sampled subset of parasites consists of 8 parasites.

To determine the co-expression relationship between *pfcrt* and other genes, we reanalyzed microarray data from our lab that profiled transcripts at 18 hr post-erythrocye invasion of 19 CQS and 17 CQR recombinant progeny of a cross between the CQR parent Dd2 and the CQS clone HB3 (GSE12515) [12]. Each gene was considered as co-expressed with *pfcrt* if the absolute Spearman correlation coefficient threshold, |*r*|, between the transcript levels of the two genes exceeded a threshold of 0.5 (|r| ≥ 0.5, FDR ≤ 0.20) across all CQR or CQS parasites. Of the 5150 genes in the *P. falciparum* genome for which transcript level data were available, transcripts for 581 (11%) genes were co-expressed with *pfcrt* in CQR progeny and 638 (12%) in CQS parasites (Figure 2 A and Additional file 2: Table S1). Of the genes that were co-expressed with *pfcrt*, 206 (30%) genes that were co-expressed with *pfcrt* in CQS parasites also were co-expressed with the gene in CQR; 70 (12%) would be expected by chance (hypergeometric test *P* = 1.25 x 10^−57^). We reasoned that if these observations are biologically meaningful, then gene pairs under high evolutionary constraint would show limited co-expression divergence in the two parasite groups. Synthetic lethal interactions are known to be under such a constraint [23,24]: in such cases, deletion of either single gene partner is compatible with growth because the second gene can buffer the loss of the first. However, the simultaneous deletion of the interacting gene leads to death. The ability of such genes to buffer mutations in their counterpart is influenced by the negative regulatory relationships that exist between them [23,25,26]. Of 14 synthetic lethal gene pairs determined by flux balance analysis [27], 2 are significantly co-expressed (Spearman correlation, |*r*| ≥ 0.5) in CQS parasites, and one of these 2 pairs also is co-expressed in CQR parasites (Additional file 1 section B and Additional file 3: Table S2). That is, when considering co-expression between synthetic lethal pairs, half of the observable co-expression relationships are conserved between the 2 networks compared to 30% in 1000 random gene pairs; however, given only two co-expressed synthetic lethal pairs, this observation is of limited value. To follow this point further, we observed that co-expressed synthetic lethal pairs in both CQS and CQR parasites are negatively correlated as expected for synthetic lethal pairs (Additional file 1: section B). No such skew towards negative correlation is observed in randomly selected gene pairs (Wilcoxon test, *P* = 0.05, Additional file 1: section B). This led us to hypothesize that, if *pfcrt* genotype constrains *pfcrt* co-expression, then the divergence of *pfcrt* co-expression networks should be much lower *within* subsets of CQS or CQR progeny than *between* the two parasite groups. The divergence between CQR and CQS progeny (Figure 2 A and B) compared to within each parasite group (Figure 2 C and D) is much higher: While only 30% of *pfcrt* co-expressed genes are similarly co-expressed between CQR and CQS progeny, this percentage rises to 57% when comparing co-expression between randomly sampled subsets of CQR or CQS (60%). The divergence within each group is not statistically significant (divergence within CQS subsets Wilcoxon test, *P* = 0.35- Figure 2 C; within CQR subsets Wilcoxon test, *P* = 0.43- Figure 2 D), while the divergence between the groups is highly significant (Wilcoxon test *P* = 6.61 × 10^−5^, Figure 2 A and B). In addition, a very strong correlation is observed between the correlations of *pfcrt* and other genes within CQR or CQS subsets (*r* = 0.99 within CQS or CQR subsets, Figure 2 C and D) compared to between CQR and CQS subsets (*r* = 0.49) (Figure 2 A and B). Together these observations indicate that different *pfcrt* genotypes are associated with functionally relevant differential co-expression.

### Co-expression partners of *pfcrt* suggest biological functions

Genes involved in related biological pathways are often co-expressed and this co-expression can result from a shared regulatory program such that co-regulated genes are co-expressed (*i.e.* correlated). As a first step to connecting genes in a transcription network, a rigorous threshold is often applied to identify non-random correlations. However, even given this initial filter, relationships between gene pairs have a strong potential to be artifacts due to intermediate/ indirect correlations (*i.e.* correlations of correlations, such that a gene may be statistically correlated with *pfcrt* by virtue of its strong correlation with another gene that is more strongly correlated with *pfcrt*). While these secondary correlations may be biologically relevant, the rate of false positives is unacceptably high for drawing functional inferences. Our TrIPI method was developed to identify and remove these third party interactions by leveraging the local network structure of gene neighborhoods to prioritize interactions (Methods, Additional file 1: section C and D). Briefly, for a given gene, *R*, TrIPI applies the triangle inequality [22] to find gene nodes potentially indirectly linking *R* to *pfcrt*. When no indirect links between *R* and *pfcrt* can be found, *R* is regarded as a functional partner of *pfcrt* and assigned a transitivity score, *t*, of zero with respect to *pfcrt*. Using this method, we found 87 high-level candidate *pfcrt* functional partners in CQS and 89 functional partners in CQR parasites (Additional file 4: Table S3). A small but significant set of 8 genes was present in both CQR and CQS (hypergeometric test *P* = 0.0001, Table 1).

**Table 1:** **Functional partners of the** ***pfcrt*** **gene that are shared between CQR and CQS co-expression networks**

**Gene ID**	**Annotation**
PF3D7_1301700	*Plasmodium* exported protein (hyp8), unknown function
PF3D7_1028000	methyltransferase, putative
PF3D7_1243600	translation initiation factor SUI1, putative
PF3D7_0814500	conserved *Plasmodium* protein, unknown function
PF3D7_0216100; PF3D7_0216200	conserved *Plasmodium* protein, unknown function
PF3D7_0501400	interspersed repeat antigen
PF3D7_1425900	conserved *Plasmodium* protein, unknown function
PF3D7_0104100	conserved *Plasmodium* membrane protein, unknown function

While *pfcrt*’s biological function is unknown, it has been proposed to be associated with the catabolism of hemoglobin-derived peptides in the parasite digestive vacuole (DV) [28,29]. These peptides are released from hemoglobin digestion by the action of proteases known as plasmepsins [30,31] and falcipains [32,33] in the DV. Once outside the DV, the peptides are broken down into amino acids for protein synthesis [34,35]. CQ interferes with the detoxification of heme [36,37] and resistance to CQ is associated with accumulation of hemoglobin derived peptides in the DV of CQR parasites, implying that hemoglobin catabolism is impaired in CQR [38].

In both CQR and CQS *pfcrt* functional partners, we observed three genes encoding hemoglobin-degrading aspartyl proteases. Two of these (plasmepsin VI- PF3D7_1033800 and plasmepsin I- PF3D7_1407900) were predicted by our method as *pfcrt* functional partners in CQR parasites, while plasmepsin IX was identified as a functional partner in CQS lines. With only 10 plasmepsin genes in *P. falciparum*, this observation is unlikely by chance (joint hypergeometric test *P* = 0.0017). The presence of distinct but functionally related hemoglobin-degrading enzymes in CQR and CQS *pfcrt* functional partners is consistent with the interpretation that this functional relationship is preserved in both co-expression networks. One explanation for this could be that the link between *pfcrt* and the plasmepsins is vital for growth supporting processes. The paralogs may present alternative routes by which parasite lines carrying different *pfcrt* alleles could mediate this essential function. We further observed other biological functions shared by *pfcrt* partners in both CQR and CQS, including heme biosynthesis and lipoamide biosynthesis, for which distinct but functionally related genes were identified as both CQR and CQS *pfcrt* functional partners. For heme biosynthesis, *pfcrt* functional partners in CQS contained the gene encoding the enzyme ferrochelatase (PF3D7_1364900), which catalyzes the terminal step in the pathway. CQR functional partners contained two genes: delta aminolevulinic acid dehydratase (PF3D7_1440300), a metabolic chokepoint in the pathway, and a putative cytochrome b5-like heme/steroid binding protein (PF3D7_0918100). Notably, heme biosynthesis occurs in the mitochondrion and the plastid organelle (apicoplast), while *pfcrt* is localized to the DV. Both of these organelles are centers for heme metabolism: heme is a byproduct of hemoglobin digestion in the DV and a synthetic metabolite in the mitochondrion.

A more surprising biological process showing functional conservation in CQR and CQS was the shared co-expression between *pfcrt* and lipoamide synthesis genes. Lipoate ligase (PF3D7_0823600) was a direct (*i.e.* predicted functional) partner of *pfcrt* in CQS and lipoamide dehydrogenase (PF3D7_1232200) was a predicted functional partner in CQR. The co-expression networks are consistent with a functional association connecting CQ, *pfcrt* and hemoglobin digestion, and also reveal novel associations that will need future validations.

### AP2 transcription factors connect *pfcrt* co-expressed genes

Because topological relationships between genes in the *pfcrt* co-expression networks can highlight potential regulators, we developed a scheme to identify candidate regulators on the basis of their connections to a large number of *pfcrt* neighbors in the network, reflecting the tendency of regulatory genes to reside in the shortest paths in networks [39,40]. The TrIPI method, in addition to identifying direct partners (*t* = 0) is also suited to identify genes with high transitivity to *pfcrt*; specifically, these are genes that provide a short path connecting *pfcrt* neighbors (Methods and Additional file 1: section D). To prioritize candidate regulators, we ranked genes by their transitivity.

Strikingly, out of 621 genes significantly correlated to *pfcrt* in CQS, the gene with the highest transitivity (*t* = 108, see Additional file 4: Table S3 for *t* values for all genes) in CQS parasites is an apicomplexan (Api) AP2 transcription factor (PF3D7_1007700), a member of the developmentally regulated ApiAP2 transcription factor family [41,42]. This gene was significantly correlated to *pfcrt* in CQS parasites (*r* = 0.53) but not in CQR (*r* = −0.1) parasites. In CQR parasites, a different AP2 transcription factor gene, PF3D7_0420300, exhibited the third highest transitivity (*r* = 0.50, *t* = 114 in CQR vs *r* = −0.09 in CQS) of 578 genes significantly correlated to *pfcrt*. Given that 27 genes in the *P. falciparum* genome encode AP2 transcription factors, the likelihood of randomly observing 2 AP2 genes within the top 3 genes from each list is extremely small (hypergeometric test, joint *P* = 0.0001). These regulators bind similar motifs [41]: the consensus motif for the second domain of the CQR AP2 is GTGTTACAC compared to GTGCAC of the third domain of the CQS AP2 [41], differing by a 3 bp central indel (TTA). It is plausible that a minor mutational event that alters their target binding specificity could facilitate a swap of regulatory programs, and lead to broad physiological and phenotypic effects mediated by their specific targets. Notably, a third AP2 (PF3D7_0802100) was predicted as a direct functional *pfcrt* partner (Additional file 4: Table S3) in CQR but not CQS parasites (CQR *r* = −0.59, *t* = 0; CQS, *r* = −0.3, FDR ≤ 0.20). In contrast to the two other AP2s, this AP2 is directly connected to *pfcrt,* implying its more restricted role within the immediate *pfcrt* co-expression network.

Because transcription factors regulate many genes, their divergent co-expression would also be expected to have pleiotropic effects. Accordingly, we developed a comprehensive gene co-expression network from an independently-derived high-dimensional transcription dataset that both validated and expanded our view of potential targets of the candidate regulators of the co-expression divergence (Methods). This network was constructed using the well-established reverse engineering algorithm, ARACNE [32], applied to a transcriptional data set obtained from 241 parasite samples cultured under a wide range of conditions [33]; from this network we computed the set of all direct links (‘regulons’) of the candidate regulators. This approach offers several advantages: it is outside of the constraints of the genetic cross and is not dependent on *pfcrt*-anchored correlations; it also provides a more general representation of genome-wide interactions using a different statistical method (mutual information rather than Spearman correlation).

The CQS-related AP2 (PF3D7_1007700) regulon contains 59 genes and is enriched for functions we observed in our *pfcrt*-based networks: hemoglobin degrading enzymes plasmepsin I (PF3D7_1407900) and falcilysin (PF3D7_1360800) as well as the heme biosynthesis enzyme delta-aminolevunilic acid (PF3D7_1440300) (Additional file 5: Table S4). The predicted regulon of the CQR AP2 contains 78 genes enriched with DNA mismatch repair (hypergeometric enrichment test *P* < 0.05, Additional file 5: Table S4).

### Candidate AP2 regulators are linked to histone acetylation

**Figure 3:**
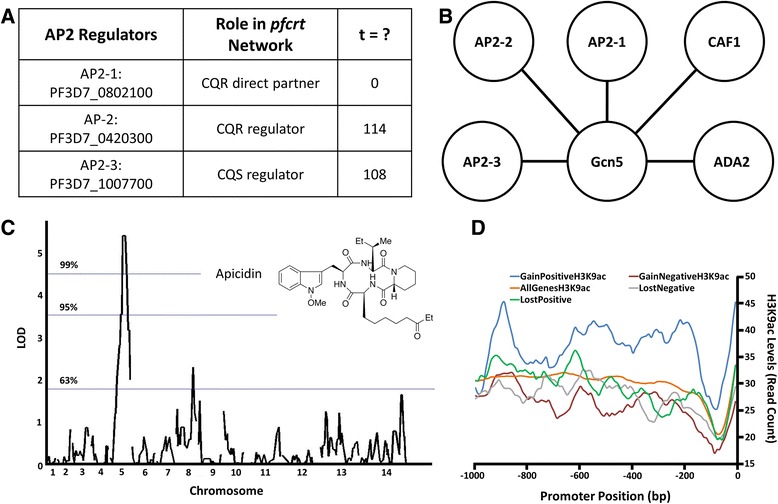
**Prediction and validation of regulatory mechanisms underlying diverging co-expression networks. (A)** Potential regulators of the *pfcrt* co-expression networks by interrogation of the topological relationships between *pfcrt* partners using the transitivity, *t*, score. Top scoring candidate regulators- the AP2 transcription factor PF3D7_1007700 (AP2-3) has the highest score in CQS while in CQR the AP2 regulator PF3D7_0420300 (AP2-2) has 3^rd^ highest score considering all genes correlated to *pfcrt* (FDR ≤ 0.20). The case of *t* =0 denotes functional (direct) *pfcrt* partners which also includes another AP2 transcription factor, PF3D7_0802100 (AP2-1). **(B)** Top scoring regulators are all part of a previously published high confidence protein-protein interaction sub-network [44] and interact with the histone acetyltransferase (Gcn5). Other transcriptional regulators physically interacting with Gcn5 include CAF1- a component of the CCR4-NOT mRNA deadenylase complex- and adenosine deaminase ADA2, leading to the hypothesis that the Gcn5 protein interaction network could be involved in integration of transcriptional regulation and mRNA stability [44]. **(C)** Validation of dysregulated histone acetylation as a potential regulatory mechanism using drug response assays. QTL mapping of quantitative dose responses to the HDACi apicidin in progeny of the Dd2 × HB3 genetic cross found significant association to genetic loci on chromosome 5, 57.3 cM (LOD = 5.4) and 8, 77.5 cM (LOD 2.3). The chromosome 5 locus includes a gene encoding CCR4 while the chromosome 8 locus contains CAF1, which physically interacts with Gcn5. **(D)** Validation of dysregulated histone acetylation using data from previous studies [53]. Promoters of the top 100 genes that are not correlated to *pfcrt* in CQS but show positive correlation in CQR (gain of positive correlation) carry vastly higher levels of H3K9ac compared to the average levels in all genes (Wilcoxon test *P* < 2.2 x 10^−16^). In contrast, H3K9ac levels of the top 100 genes that gain negative correlation are significantly lower compared to the genome-wide promoter baseline (Wilcoxon test *P* = 3.4 x 10^−16^).

The divergent co-expression of 3 AP2 transcription factor genes in the *pfcrt* co-expression networks led us to investigate their functional relationships (Figure 3 A and B). While limited information is available about these regulators (PF3D7_0802100, PF3D7_0420300 and PF3D7_1007700), [41-43], we were intrigued to note that LaCount et al. [44] identified physical interactions among them as part of a high confidence protein-protein interaction subnetwork centered on the histone acetyltransferase, *Gcn5*, and containing additional chromatin-modifying proteins (Figure 3 B) [44]. The interactions among these transcription factors and *Gcn5*-containing complexes suggest that the AP2s interface with histone acetylation in their regulatory roles. These interactions also have been observed in *Toxoplasma* [45] and *Arabidopsis* [46]. That these associations may extend to regulatory relationships is implied by the up-regulation of the AP2 genes following perturbations by apicidin, a histone deacetylase inhibitor (HDACi) [47]. Consistent with these observations, the predicted regulon of the AP2 in the CQS *pfcrt* network (PF3D7_1007700) includes *Gcn5*, *CCR4* and two hypothetical proteins (PF3D7_1366900 and PF3D7_0817300) that are also members of the *Gcn5* protein-protein interaction sub-network [44]. Moreover, the *Gcn5* regulon contains *ADA2* and *CCR4* associated factor 1 (*CAF1*) which are integral components of the *CCR4-NOT* complex, a regulator of mRNA stability and transcriptional regulation [48-51].

We hypothesized that the association between the CQS AP2 (PF3D7_1007700) and *Gcn5*, found in both the protein-protein interaction complex (Figure 3 B) and in their regulons that were constructed independent of the protein-protein network, predicts that dys-regulation of histone acetylation in CQR parasites. This dysregulation could manifest as differential sensitivity to perturbations by histone acetylation/ deacetylation inhibitors. To test this idea, we performed quantitative dose response assays on the CQR and CQS recombinant progeny clones using the HDACi- apicidin. The parasites exhibited a range of sensitivities (Additional file 6: Table S5) and QTL analysis (Figure 3 C) provided insights into the molecular basis of this differential sensitivity. Two genetic loci, on chromosome 5 (cM 57.3; LOD = 5.4, chi-square *P* = 0.000001, genome-wide significance threshold *P* < 0.01 [52]) and a suggestive peak on chromosome 8 (cM 77.5; LOD = 2.3, chi-square *P* = 0.001120, genome-wide significance threshold *P* < 0.37) are associated with the level of apicidin response (Figure 3 C). Genes lying between genetic markers flanking the 95% confidence range of each QTL peak were regarded as potential candidates.

The chromosome 5 locus contains a putative *CCR4* gene and the chromosome 8 locus includes the *CAF1* gene. *CAF1* physically interacts with both *Gcn5* and the AP2 predicted to be a functional partner of *pfcrt* in CQR (PF3D7_0802100) [44]; it resides within a protein-protein interaction complex that includes the two regulatory candidates identified in our networks (the AP2s PF3D7_1007700 and PF3D7_0420300) (Figure 3 A and B). These data point to a possible role of these AP2s in dysregulation of the *Gcn5* network, resulting into an altered response to HDACi. A better understanding of how these AP2s interface with histone acetylation will require functional assays of the interactions between these components that differ in CQR and CQS parasites.

### Promoters of differentially co-expressed genes have distinct epigenetic profiles

The physical interactions between the AP2 regulators with *Gcn5* coupled to the heritable variation in dose response to apicidin (Figure 3 C) collectively imply divergent histone acetylation in the recombinant progeny clones. Therefore, we hypothesized that differentially co-expressed *pfcrt* interacting genes between CQR and CQS parasites would differ in histone acetylation patterns. To test this, we partitioned genes into categories of extreme differential co-expression in CQR vs CQS. Specifically, we identified genes that gained or lost correlations (positive or negative) with *pfcrt* in CQR parasites relative to CQS. We then asked whether the promoters of genes that lost or gained correlation with *pfcrt* exhibited distinct histone acetylation profiles. For each category, we considered the top 100 genes and examined histone acetylation patterns in the 1000 bp upstream of translation start site using a publicly available dataset [53]. Genes that gained positive correlation had higher levels of the acetylated histone H3K9ac in their promoters while those that gained negative correlation had lower levels of this histone modification compared to the genome baseline (Figure 3 D, Wilcoxon test *P* < 0.05). Promoters of genes that lost positive correlation were no different from the genome baseline (Figure 3 D, Wilcoxon test *P* = 0.27) while those that lost negative correlation were significantly different from the baseline, particularly in the -200 to -500 bp upstream region (Figure 3D, Wilcoxon test *P* = 1.2 × 10^−11^). These data further support our conclusion that the CQR and CQS progeny have divergent regulatory processes involving transcription factors and epigenetic modifications.

### Divergent co-expression networks predict diverging phenotypes

We reasoned that co-expression networks, particularly the genes with marked differential co-expression between CQR and CQS could predict additional phenotypic differences between the two groups of recombinant progeny, as was observed for the QTL associated with apicidin dose response (Figure 3 C and Additional file 1: section E). Besides differences in CQ susceptibility, recombinants of the Dd2 × HB3 genetic cross differ for other phenotypes. For example, genetic association mapping of quantitative dose responses showed that the *pfcrt* locus is associated with response to more than 200 compounds [54]. Other studies suggest that the H^+^ physiology of the DV is altered in CQR, although how this affects compartmental pH [55] and vacuolar volume [56] are still debated [57,58]. Evidence suggests that, in CQR parasites, CQ effluxes from the DV in conjunction with H^+^ via a verapamil-sensitive pathway [59].

An altered co-expression network could account for some of these unexplained broader phenotypic changes associated with the *pfcrt* mutation. In CQR but not CQS networks, components of pH regulation were specifically enriched in the *pfcrt* functional partners (hypergeometric test *P* = 0.004, Additional file 4: Table S3). For example, transcript levels of the V-type H^+^ translocating pyrophosphatase (PF3D7_1456800), involved in pH regulation was a functional partner of *pfcrt* (*r* = 0.61, FDR ≤ 0.20, *t* = 0) in CQR but not CQS progeny. Co-expression of the two genes could compensate for the loss of proton levels during CQ efflux in CQR strains [59] by increasing influx of hydrogen ions into the DV.

### Altered co-expression networks are associated with MMR dysregulation in CQR parasites

As outlined above (and additional details in Additional file 1: section E), co-expression changes can compromise normal functional associations between genes, thereby altering cellular response to secondary perturbations and exposing drug unique susceptibilities specifically in CQR parasites. For example, the co-expression constraint described for synthetic lethal gene pairs implies that dysregulation of their coordinated expression is incompatible with their normal ability to compensate for each other [25,60] (Additional file 1: section E). A well-documented case of this effect involves the synthetic lethal gene pair *tbx-8* and *tbx-9* in *Caenorhabditis elegans* [61,62]. Deletion of *tbx-9* results in an incompletely penetrant phenotype in which individuals expressing high levels of *tbx-8* exhibit the wild-type phenotype, while those expressing low levels produce an abnormal phenotype [26]. High expression of *tbx-8* buffers deletion of *tbx-9*. This buffering effect is lost in individuals expressing low levels of *tbx-8*, making these individuals vulnerable to disruption of *tbx-9* [26].

**Figure 4:**
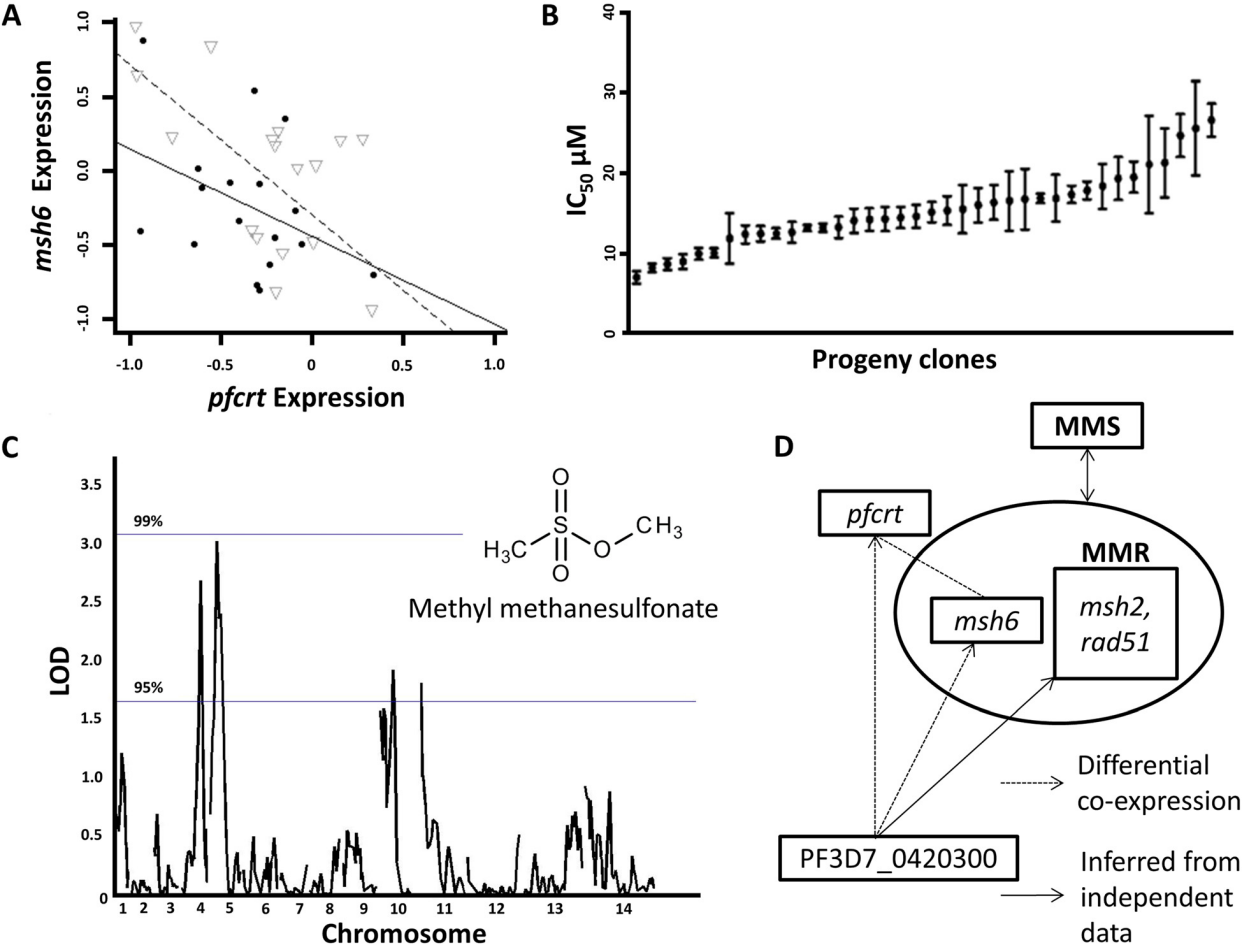
**Validation of dysregulated DNA mismatch repair (MMR) pathway. (A)** Divergent co-expression of *pfcrt* and the *msh6* gene in CQR versus CQS parasites. Each dot or triangle represents the transcript level of the 2 genes in CQR and CQS parasites, respectively. The solid line is a linear fit of the data from CQR parasites and the dotted line is for data from CQS parasites. **(B)** Quantitative dose response variation in the response to the DNA damaging agent MMS across the Dd2 × HB3 genetic cross. **(C)** QTL mapping results for the MMS dose response reveals two candidate loci- chromosome 4, 60.3 cM (LOD = 2.68, chi-square *P* = 0.00044) and 5, 20 cM (LOD = 3.02, chi-square *P* = 0.000194). **(D)** The predicted regulon of the candidate regulator AP2 in CQR (PF3D7_0420300) is enriched with DNA repair genes including *msh2* and *rad51*, providing further validation of MMR dysregulation. Prediction of potential targets of this AP2 was performed using an independent transcriptional data set as described in methods [68].

We searched the functional partners of *pfcrt* having an altered co-expression in CQR vs CQS for genes encoding druggable proteins or genes in pathways associated with antimalarial drug resistance. The mismatch repair (MMR) pathway meets these criteria: the gene encoding the DNA MMR protein (*msh6*, PF3D7_0505500) is a functional partner of *pfcrt* in CQS but not CQR parasites (Figure 4 A, correlation coefficient between *pfcrt* and *msh6* for CQS, *r* = −0.6 and for CQR, *r* = −0.3, FDR ≤ 0.20) and this pathway can be induced by small molecules such as methyl methanesulfonate (MMS) [60]. We predicted that the CQR genetic background may be differentially susceptible to perturbations of this pathway. To test this prediction, we performed quantitative dose–response assays to MMS [63] and found that the parental line Dd2 and the CQS line HB3 were similarly sensitive to growth inhibition by MMS, but their progeny clones exhibited a range of susceptibility to the drug (Figure 4 B, Additional file 6: Table S5). QTL mapping of the quantitative susceptibility to MMS of the progeny identified genetic loci on chromosomes 4, 60.3 cM (LOD = 2.68) and 5, 20 cM (LOD = 3.02) (Figure 4 C). Suggestive peaks also occurred at chromosome 10, 40.2 cM (LOD = 1.92) and 11, 23 cM (LOD = 1.8).

Importantly, *msh6* resides within the physical location of the chromosome 5 QTL (Figure 4 C, chi-square *P* = 0.00044). Strikingly, the chromosome 4 QTL includes the AP2 transcription factor gene which we predicted as a regulator of the CQR *pfcrt* co-expression network (chi-square *P* = 0.000194). A closer examination of the predicted targets of this transcription factor (its regulon, see methods) identifies 78 genes functionally enriched for DNA repair (hypergeometric test, *P* < 0.02, Additional file 5: Table S4). Target genes of this transcription factor with a direct role in DNA repair include an *msh2* homolog (PF3D7_1427500), *Rad51* homolog (PF3D7_1107400), deoxyuridine 5'-triphosphate nucleotidylhydrolase (PF3D7_1127100), nucleoside diphosphate kinase (PF3D7_1366500) and DNA topoisomerase II (PF3D7_1433500). This independently-derived network strongly supports the prediction that this AP2 transcription factor drives the connection between divergent *pfcrt* co-expression and MMS response (Figure 4 D).

The association between genetic loci encoding the predicted AP2 regulator of the *pfcrt* co-expression network, PF3D7_0420300, and the divergently co-expressed *msh6* with MMS response highlights the value of a generalized application of divergent co-expression networks for predicting phenotypic outcomes, for example, drug responses of specific groups of parasites.

## Discussion

We constructed *pfcrt* co-expression networks that differ between CQR and CQS parasites and mined these networks to show that: i) consistently co-expressed gene sets across the recombinant progeny clones of both drug resistance states reflect core shared functions, chiefly hemoglobin metabolism, in line with proposed *pfcrt* function, CQ modes of action and resistance, ii) the topologies of these 2 co-expression networks are held together by 2 transcriptional regulators from the same family (AP2), possibly interfacing with histone acetylation (Figure 3), iii) divergent co-expression networks can be useful predictors of phenotypic divergence, as demonstrated here by the association between the genetic loci encoding the divergent processes (DNA repair and histone acetylation) and dose response variation to small molecules targeting these processes (MMS and apicidin, Figure 3 C and 4 C).

Our results indicate that co-expression networks can generate extensive but cryptic variation. Such variation would be undetectable by genome sequencing, the principal method used to track parasite evolution and emergence of drug resistance. The dysregulation of regulatory networks can affect multiple target genes, thereby providing a molecular explanation for broad (pleiotropic) phenotypic differences. We predicted and validated that dysregulation of DNA repair is associated with divergent co-expression of *msh6*. Interestingly, the CQR parental clone Dd2 was derived from an Indochina clone, W2, which exhibits an accelerated resistance to multiple drugs (ARMD) in laboratory experiments [64]. Given that co-expression relationships are known to buffer deleterious mutations [25,26,60,65], we propose that differential co-expression of genes associated with drug response can expose drug resistant parasites to susceptibilities to specific drugs (Additional file 1: section E).

We envision that the regulatory divergence between CQR and CQS could involve at least 2 mechanisms (Additional file 1: section F): i) a regulon swap in which the mutant *pfcrt* gene is recruited into a new regulatory program by cis-mutations or by alterations of histone acetylation marks in its promoter (Additional file 1: Figure S5A), and/or ii) a physiologically mediated mechanism in which mutant *pfcrt* has an additional metabolic effect, for example the verapamil sensitive H^+^ associated leak [59], that could activate downstream transcriptional regulators (Additional file 1: Figure S5B). Genome sequence data of the progeny clones combined with histone modification data sets will be important in experimentally validating the regulatory basis of the co-expression divergence in CQR and CQS parasites.

Our observations are constrained by the fact that all datasets used in this study were obtained from recombinant progeny clones from a single genetic cross. Therefore, the genetic diversity observed in this data set does not fully capture the diversity of CQR and CQS parasites in the field. Further studies using field isolates will be crucial in leveraging and extending co-expression networks to understand molecular changes associated with drug resistance, particularly the devastating emergence of artemisinin resistance in Southeast Asia [66] and other phenotypic states for which the primary causal genes are known.

## Conclusion

We have shown that *pfcrt* is differentially co-expressed in CQR parasites as compared to CQS parasites. This differential co-expression reveals biological pathways that are divergent in CQR parasites and regulatory processes underlying the differential co-expression. We validate that two key biological processes associated with the differentially co-expressed *pfcrt* partners – DNA MMR pathway and histone acetylation- are linked to differential responses to small molecules targeting these processes. We conclude that co-expression divergence can complement traditional genetic and molecular approaches to uncovering the molecular basis of phenotypic divergence as well as predicting phenotypic outcomes.

## Methods

### Construction of co-expression networks

Given transcript data at 18 hr post-invasion time-point from 17 CQR and 19 CQS parasite clones derived from the Dd2 × HB3 genetic cross [12], GSE12515, we computed the Spearman rank correlation, *r*, between probes representing the *pfcrt* gene to every other gene.

For each gene, correlation coefficients from each probe were then used to compute an average correlation between the gene and *pfcrt*. An absolute correlation coefficient of 0.5 was regarded as significant (Benjamini-Hochberg FDR ≤ 0.20). The FDR was determined as follows. For both CQR and CQS gene expression data we computed the Spearman rank correlation, *r*, and a corresponding *P*-value using a t-statistic between each *pfcrt* probe to probes of each of the other genes. An absolute correlation co-efficient cut-off of 0.5 was then chosen. This corresponds to a False Discovery Rate (FDR) of 0.20 as calculated by the Benjamini-Hochberg method [67]. In order to detect co-expression divergence, we considered both the sign and magnitude of correlation between each transcript and *pfcrt* in CQS vs CQR. A transcript was considered to gain a correlation to *pfcrt* if the correlation in CQR but not CQS was significant and a loss of correlation if the correlation was significant in CQS but not CQR strains.

### Co-expression analysis of synthetic lethal gene pairs

Synthetic lethal gene pairs were obtained from published flux-balance analysis simulations [27]. Spearman correlation was then computed for each of the synthetic lethal gene pairs. A background distribution for Spearman correlations between gene pairs was obtained by randomly sampling 1000 gene pairs and computing correlations between them. To compare the correlation distributions in synthetic lethal gene pairs vs random gene pairs, probability density plots were generated and compared using Wilcoxon test.

### Co-expression divergence between CQR and CQS vs within the groups

To compare *pfcrt* co-expression divergence between subsets of CQR and CQS parasites, we randomly sampled 8 CQR and 8 CQS parasites. This subsampling procedure was repeated 100 times to obtain 100 pairs of randomly sampled subsets of CQR and CQS parasites. For each pair of the subsets, we determined the correlation between *pfcrt* transcript levels to that of all other genes and regarded genes with an absolute Spearman correlation coefficient ≥ 0.5 as correlated to *pfcrt*. For each gene, we compared its correlation to *pfcrt* in each of the pairs of subsets and determined the number of times it shows significant correlation to *pfcrt* in the pair *i.e.* conserved between subsets of CQR and CQS parasites. To determine the co-expression divergence within subsets of CQR parasites, we repeated the same procedure in 100 random pairs of CQR parasite subsets. A similar process was performed to compare co-expression divergence within subsets of CQS parasites.

### Prediction of *pfcrt* functional and regulatory candidates

All genes significantly correlated to *pfcrt* (FDR ≤ 0.20) were further prioritized into functional partners using TrIPI (Additional file 1: Figure S2). A correlation matrix containing correlations between all pairs of genes significantly correlated to *pfcrt* was generated. The association between *pfcrt* and any given gene say *R*, was then accessed in relation to each of the other remaining set of genes, *S*, using triangle inequality. Genes with *t* = 0 were regarded as functional partners of *pfcrt* since the correlation between such genes and *pfcrt* cannot be accounted for by transitive correlations. On the other hand, genes with the highest *t* represent highly connected genes in the neighborhood of *pfcrt* and are potential regulatory candidates

### Prediction of transcription factor regulons

Regulatory targets of the AP2 transcription factors were predicted using independent transcriptional dataset consisting of 241 arrays from multiple drug perturbations for 3 different parasite strains [68] and the reverse engineering algorithm ARACNE [69]. Briefly, we obtained 100 bootstrap samples of the transcriptional data and in each bootstrap used ARACNE to compute the mutual information between transcript levels of the AP2 and all other genes. Potential false positives were pruned using a data processing inequality (DPI) value of 0.15 and a *P* < 10^−5^. Only interactions predicted in multiple bootstrap runs were retained (*P* < 10^−5^). Biological function enrichments were performed using a hypergeometric test implemented on the MADIBA webserver [70]. Biological function categories were regarded as enriched at a corrected FDR of 0.05.

### Promoter histone acetylation analysis

The histone acetylation levels of the promoters of gene sets were obtained from a recent study [53], GSE2387. The 1000 bp sequence upstream of the translation start of genes was considered as the promoter region and data representing the read coverage from ChIP-seq following immuno-precipitation by antibodies against Ty1-tagged version of H3K9ac antibodies were taken to reflect the modified H3K9ac at 20 hrs post invasion [53]. Promoter analysis considered the top 100 genes whose co-expression to *pfcrt* was highly divergent between CQR and CQS parasites. Gene categories were defined as gain (positive or negative) or loss (positive or negative) of correlation with respect to their correlation to *pfcrt* in CQR vs CQS based on data provided in Additional file 2: Table S1. Promoter read coverage obtained from sequencing of the H3K9ac pull down was averaged across promoters of each of the gene categories for comparisons. H3K9ac level between each category of genes to the baseline (average across all promoters in the genome) was obtained using a t-test.

### Validations using dose response assays and QTL

Parasite clones were cultured using standard methods in human red blood cells (Indiana Regional Blood Center, Indianapolis, Indiana) suspended in complete medium containing RPMI 1640 with L-glutamine (Invitrogen Corp.), 50 mg/L hypoxanthine (Sigma-Aldrich), 25 mM HEPES (Cal Biochem), 0.5% Albumax II (Invitrogen Corp.), 10 mg/L gentamicin (Invitrogen Corp.) and 0.225% NaHCO_3_ (Biosource)] at 5% hematocrit. Cultures were grown separately in sealed flasks at 37 °C under an atmosphere of 5% CO_2_, 5% O_2_, and 90% N_2_. Dose response assays were performed as previously described [71]. QTL analysis for the dose responses in the Dd2 × HB3 genetic cross was performed using previously published statistical methods in Pseudomarker Version 2.04 [72]. The statistical significance of the obtained log odds scores (LOD) were obtained from a chi-square distribution, *P* = 1 - chi2cdf(2 × LOD score × Log_10_,degree of freedom = 1) where chi2cdf is the Matlab chi-square cumulative distribution function. The genome-wide significance thresholds were computed using permutation analysis as described by Doerge and Churchill [52] and implemented in Pseudomarker [72] (genome-wide adjusted *P* < 0.37, suggestive; *P* < 0.05, significant; *P* < 0.01, highly significant).

## Additional files

Additional file 1:
**Section A-F Supplementary text and figures.**
Additional file 2: Table S1. Spearman correlation coefficients between *pfcrt* and other genes at a threshold of FDR ≤ 0.20, |*r*| = 0.5) in CQR or CQS. Additional file 3: Table S2. Spearman correlation coefficients between synthetic lethal gene pairs. Additional file 4: Table S3. Transitivity scores for genes significantly correlated to *pfcrt* in CQS or CQR parasites. GO enrichment analysis of *pfcrt* functional partners. Additional file 5: Table S4. Predicted regulons of the candidate AP2 regulators for CQS and CQR co-expression networks. Additional file 6: Table S5. Dose response data for apicidin and MMS in the Dd2 × HB3 genetic cross.

## References

[1] Vitkup D, Kharchenko P, Wagner A (2006). Influence of metabolic network structure and function on enzyme evolution. Genome Biol.

[2] Nadeau JH (2001). Modifier genes in mice and humans. Nat Rev Genet.

[3] Seidel HS, Rockman MV, Kruglyak L (2008). Widespread genetic incompatibility in C. elegans maintained by balancing selection. Science.

[4] Romeo G, McKusick VA (1994). Phenotypic diversity, allelic series and modifier genes. Nat Genet.

[5] Scriver CR, Waters PJ (1999). Monogenic traits are not simple: lessons from phenylketonuria. Trends Genet.

[6] Dipple KM, McCabe ER (2000). Phenotypes of patients with "simple" Mendelian disorders are complex traits: thresholds, modifiers, and systems dynamics. Am J Hum Genet.

[7] Sidhu AB, Verdier-Pinard D, Fidock DA (2002). Chloroquine resistance in Plasmodium falciparum malaria parasites conferred by pfcrt mutations. Science.

[8] Fidock DA, Nomura T, Talley AK, Cooper RA, Dzekunov SM, Ferdig MT (2000). Mutations in the P. falciparum digestive vacuole transmembrane protein PfCRT and evidence for their role in chloroquine resistance. Mol Cell.

[9] Cooper RA, Hartwig CL, Ferdig MT (2005). pfcrt is more than the Plasmodium falciparum chloroquine resistance gene: a functional and evolutionary perspective. Acta Trop.

[10] Wellems TE, Walker-Jonah A, Panton LJ (1991). Genetic mapping of the chloroquine-resistance locus on Plasmodium falciparum chromosome 7. Proc Natl Acad Sci U S A.

[11] Cooper RA, Ferdig MT, Su XZ, Ursos LM, Mu J, Nomura T (2002). Alternative mutations at position 76 of the vacuolar transmembrane protein PfCRT are associated with chloroquine resistance and unique stereospecific quinine and quinidine responses in Plasmodium falciparum. Mol Pharmacol.

[12] Gonzales JM, Patel JJ, Ponmee N, Jiang L, Tan A, Maher SP (2008). Regulatory hotspots in the malaria parasite genome dictate transcriptional variation. PLoS Biol.

[13] van Noort V, Snel B, Huynen MA (2003). Predicting gene function by conserved co-expression. Trends Genet.

[14] Oldham MC, Horvath S, Geschwind DH (2006). Conservation and evolution of gene coexpression networks in human and chimpanzee brains. Proc Natl Acad Sci U S A.

[15] Kostka D, Spang R (2004). Finding disease specific alterations in the co-expression of genes. Bioinformatics.

[16] Penrod NM, Moore JH (2014). Influence networks based on coexpression improve drug target discovery for the development of novel cancer therapeutics. BMC Syst Biol.

[17] Penrod NM, Moore JH (2013). Key genes for modulating information flow play a temporal role as breast tumor coexpression networks are dynamically rewired by letrozole. BMC Med Genomics.

[18] de la Fuente A (2010). From 'differential expression' to 'differential networking' - identification of dysfunctional regulatory networks in diseases. Trends Genet.

[19] Carter SL, Brechbuhler CM, Griffin M, Bond AT (2004). Gene co-expression network topology provides a framework for molecular characterization of cellular state. Bioinformatics.

[20] Hudson NJ, Reverter A, Dalrymple BP (2009). A differential wiring analysis of expression data correctly identifies the gene containing the causal mutation. PLoS Comput Biol.

[21] Hudson NJ, Dalrymple BP, Reverter A (2012). Beyond differential expression: the quest for causal mutations and effector molecules. BMC Genomics.

[22] Rice JJ, Tu Y, Stolovitzky G (2005). Reconstructing biological networks using conditional correlation analysis. Bioinformatics.

[23] Folger O, Jerby L, Frezza C, Gottlieb E, Ruppin E, Shlomi T (2011). Predicting selective drug targets in cancer through metabolic networks. Mol Syst Biol.

[24] Park S, Lehner B (2013). Epigenetic epistatic interactions constrain the evolution of gene expression. Mol Syst Biol.

[25] DeLuna A, Springer M, Kirschner MW, Kishony R (2010). Need-based up-regulation of protein levels in response to deletion of their duplicate genes. PLoS Biol.

[26] Burga A, Casanueva MO, Lehner B (2011). Predicting mutation outcome from early stochastic variation in genetic interaction partners. Nature.

[27] Plata G, Hsiao TL, Olszewski KL, Llinas M, Vitkup D (2010). Reconstruction and flux-balance analysis of the Plasmodium falciparum metabolic network. Mol Syst Biol.

[28] Martin RE, Marchetti RV, Cowan AI, Howitt SM, Broer S, Kirk K (2009). Chloroquine transport via the malaria parasite's chloroquine resistance transporter. Science.

[29] Martin RE, Kirk K (2004). The malaria parasite's chloroquine resistance transporter is a member of the drug/metabolite transporter superfamily. Mol Biol Evol.

[30] Banerjee R, Liu J, Beatty W, Pelosof L, Klemba M, Goldberg DE (2002). Four plasmepsins are active in the Plasmodium falciparum food vacuole, including a protease with an active-site histidine. Proc Natl Acad Sci U S A.

[31] Liu J, Istvan ES, Gluzman IY, Gross J, Goldberg DE (2006). Plasmodium falciparum ensures its amino acid supply with multiple acquisition pathways and redundant proteolytic enzyme systems. Proc Natl Acad Sci U S A.

[32] Shenai BR, Sijwali PS, Singh A, Rosenthal PJ (2000). Characterization of native and recombinant falcipain-2, a principal trophozoite cysteine protease and essential hemoglobinase of Plasmodium falciparum. J Biol Chem.

[33] Singh N, Sijwali PS, Pandey KC, Rosenthal PJ (2006). Plasmodium falciparum: biochemical characterization of the cysteine protease falcipain-2'. Exp Parasitol.

[34] Dalal S, Klemba M (2007). Roles for two aminopeptidases in vacuolar hemoglobin catabolism in Plasmodium falciparum. J Biol Chem.

[35] Kolakovich KA, Gluzman IY, Duffin KL, Goldberg DE (1997). Generation of hemoglobin peptides in the acidic digestive vacuole of Plasmodium falciparum implicates peptide transport in amino acid production. Mol Biochem Parasitol.

[36] Wellems TE (1992). Malaria. How chloroquine works. Nature.

[37] Slater AF, Cerami A (1992). Inhibition by chloroquine of a novel haem polymerase enzyme activity in malaria trophozoites. Nature.

[38] Lewis IA, Wacker M, Olszewski KL, Cobbold SA, Baska KS, Tan A (2014). Metabolic QTL analysis links chloroquine resistance in plasmodium falciparum to impaired hemoglobin catabolism. PLoS Genet.

[39] Tu Z, Wang L, Arbeitman MN, Chen T, Sun F (2006). An integrative approach for causal gene identification and gene regulatory pathway inference. Bioinformatics.

[40] Shih YK, Parthasarathy S (2012). A single source k-shortest paths algorithm to infer regulatory pathways in a gene network. Bioinformatics.

[41] Campbell TL, De Silva EK, Olszewski KL, Elemento O, Llinas M (2010). Identification and genome-wide prediction of DNA binding specificities for the ApiAP2 family of regulators from the malaria parasite. PLoS Pathog.

[42] Balaji S, Babu MM, Iyer LM, Aravind L (2005). Discovery of the principal specific transcription factors of Apicomplexa and their implication for the evolution of the AP2-integrase DNA binding domains. Nucleic Acids Res.

[43] Painter HJ, Campbell TL, Llinas M (2011). The Apicomplexan AP2 family: integral factors regulating Plasmodium development. Mol Biochem Parasitol.

[44] LaCount DJ, Vignali M, Chettier R, Phansalkar A, Bell R, Hesselberth JR (2005). A protein interaction network of the malaria parasite Plasmodium falciparum. Nature.

[45] Dixon SE, Stilger KL, Elias EV, Naguleswaran A, Sullivan WJ (2010). A decade of epigenetic research in Toxoplasma gondii. Mol Biochem Parasitol.

[46] Stockinger EJ, Gilmour SJ, Thomashow MF (1997). Arabidopsis thaliana CBF1 encodes an AP2 domain-containing transcriptional activator that binds to the C-repeat/DRE, a cis-acting DNA regulatory element that stimulates transcription in response to low temperature and water deficit. Proc Natl Acad Sci U S A.

[47] Chaal BK, Gupta AP, Wastuwidyaningtyas BD, Luah YH, Bozdech Z (2010). Histone deacetylases play a major role in the transcriptional regulation of the Plasmodium falciparum life cycle. PLoS Pathog.

[48] Dori-Bachash M, Shema E, Tirosh I (2011). Coupled evolution of transcription and mRNA degradation. PLoS Biol.

[49] Collart MA (2003). Global control of gene expression in yeast by the Ccr4-Not complex. Gene.

[50] Denis CL, Chen J (2003). The CCR4-NOT complex plays diverse roles in mRNA metabolism. Prog Nucleic Acid Res Mol Biol.

[51] Balu B, Maher SP, Pance A, Chauhan C, Naumov AV, Andrews RM (2011). CCR4-associated factor 1 coordinates the expression of Plasmodium falciparum egress and invasion proteins. Eukaryot Cell.

[52] Churchill GA, Doerge RW (1994). Empirical threshold values for quantitative trait mapping. Genetics.

[53] Bartfai R, Hoeijmakers WA, Salcedo Amaya AM, Smits AH, Janssen Megens E, Kaan A (2010). H2A.Z demarcates intergenic regions of the plasmodium falciparum epigenome that are dynamically marked by H3K9ac and H3K4me3. PLoS Pathog.

[54] Yuan J, Cheng KC, Johnson RL, Huang R, Pattaradilokrat S, Liu A (2011). Chemical genomic profiling for antimalarial therapies, response signatures, and molecular targets. Science.

[55] Bennett TN, Kosar AD, Ursos LM, Dzekunov S, Singh Sidhu AB, Fidock DA (2004). Drug resistance-associated pfCRT mutations confer decreased Plasmodium falciparum digestive vacuolar pH. Mol Biochem Parasitol.

[56] Gligorijevic B, Bennett T, McAllister R, Urbach JS, Roepe PD (2006). Spinning disk confocal microscopy of live, intraerythrocytic malarial parasites. 2. Altered vacuolar volume regulation in drug resistant malaria. Biochemistry.

[57] Hayward R, Saliba KJ, Kirk K (2006). The pH of the digestive vacuole of Plasmodium falciparum is not associated with chloroquine resistance. J Cell Sci.

[58] Kirk K, Saliba KJ (2001). Chloroquine resistance and the pH of the malaria parasite's digestive vacuole. Drug Resist Updat.

[59] Lehane AM, Hayward R, Saliba KJ, Kirk K (2008). A verapamil-sensitive chloroquine-associated H+ leak from the digestive vacuole in chloroquine-resistant malaria parasites. J Cell Sci.

[60] DeLuna A, Vetsigian K, Shoresh N, Hegreness M, Colon-Gonzalez M, Chao S (2008). Exposing the fitness contribution of duplicated genes. Nat Genet.

[61] Andachi Y (2004). Caenorhabditis elegans T-box genes tbx-9 and tbx-8 are required for formation of hypodermis and body-wall muscle in embryogenesis. Genes Cells.

[62] Baugh LR, Wen JC, Hill AA, Slonim DK, Brown EL, Hunter CP (2005). Synthetic lethal analysis of Caenorhabditis elegans posterior embryonic patterning genes identifies conserved genetic interactions. Genome Biol.

[63] Glaab WE, Tindall KR, Skopek TR (1999). Specificity of mutations induced by methyl methanesulfonate in mismatch repair-deficient human cancer cell lines. Mutat Res.

[64] Rathod PK, McErlean T, Lee PC (1997). Variations in frequencies of drug resistance in Plasmodium falciparum. Proc Natl Acad Sci U S A.

[65] Casanueva MO, Burga A, Lehner B (2012). Fitness trade-offs and environmentally induced mutation buffering in isogenic C. elegans. Science.

[66] Na-Bangchang K, Karbwang J (2013). Emerging artemisinin resistance in the border areas of Thailand. Expert Rev Clin Pharmacol.

[67] Benjamini Y, Hochberg Y (1995). Controlling the false discovery rate: a practical and powerful approach to multiple testing. J R Stat Soc Ser B.

[68] Hu G, Cabrera A, Kono M, Mok S, Chaal BK, Haase S (2010). Transcriptional profiling of growth perturbations of the human malaria parasite Plasmodium falciparum. Nat Biotechnol.

[69] Basso K, Margolin AA, Stolovitzky G, Klein U, Dalla-Favera R, Califano A (2005). Reverse engineering of regulatory networks in human B cells. Nat Genet.

[70] Law PJ, Claudel Renard C, Joubert F, Louw AI, Berger DK (2008). MADIBA: a web server toolkit for biological interpretation of Plasmodium and plant gene clusters. BMC Genomics.

[71] Ferdig MT, Cooper RA, Mu J, Deng B, Joy DA, Su XZ (2004). Dissecting the loci of low-level quinine resistance in malaria parasites. Mol Microbiol.

[72] Sen S, Churchill GA (2001). A statistical framework for quantitative trait mapping. Genetics.

