# Supplementary material for: Predicting functional and regulatory divergence of a drug resistance transporter gene in the human malaria parasite
Source: BMC Genomics. 2015 Feb 22;16(1):115. doi: 10.1186/s12864-015-1261-6 (PMC4352545; doi:10.1186/s12864-015-1261-6)
Supplement: Additional file 1: — Section A-F Supplementary text and figures. [file 12864_2015_1261_MOESM1_ESM.pdf]

Supplementary Text

A. Comprehensive overview of the approach

The approach presented in this paper seeks to determine *pfcrt* interacting genes and to use the co-expression patterns to gain insights into 3 main questions: i) what are the biological functions of *pfcrt*?, ii) what are the diverging biological processes in chloroquine resistant (CQR) parasites relative to their sensitive (CQS) counterparts?, and iii) what are the regulatory processes mediating the different co-expression patterns? The co-expression networks constructed in this study are hypothesis-driven (as specifically anchored on *pfcrt*) and constructed separately for CQR and CQS parasites. This is a departure from the typical case in which pairwise co-expression relationships among all genes are used to construct a network. The approach can be generally applied to investigate biological functions and regulatory mechanisms that differ between 2 phenotypic states- here CQR and CQS- and for which a causal gene mutation is known.

The rigor of our method comes from the sequential steps performed using transcriptional data of many individuals from each phenotypic state (Figure 1): i) Spearman correlations are computed between transcript levels of the causal gene in this case, *pfcrt*) and all other genes in the genome (Figure 2 A). An initial threshold of correlations is then taken at a permutation-based FDR = 0.05. ii) Genes that pass the initial threshold are subjected to further prioritization into predicted functional partners by applying the triangle inequality to prune potential indirect edges (see Methods and Supplementary Figure 2). iii) Regulatory candidates for controlling co-expression divergence are also predicted by leveraging triangle inequality to identify genes that

provide the shortest paths connecting the predicted functional partners of the causal gene (Methods and Supplementary Figure 3). Transcription factor genes that are ranked highly amongst regulatory candidates are selected for experimental validations (Figure 3 A and B). Regulatory candidates are also corroborated using independent data sets as follows. First, independent protein-protein interaction data are used to identify functional interactions among the regulators and the context of their regulatory roles (Figure 3 C). Second, promoters of divergently co-expressed genes are examined for divergent regulatory characteristics indicated by members of the protein-protein networks (histone acetylation in this study). Third, a comprehensive independent transcriptional network is scanned for predicted targets of the candidate regulators contain genes that are divergently co-expressed.

Finally, experimental validation of divergent functional and regulatory processes is conducted by profiling the dose responses of CQR and CQS parasites to small molecules that affect the divergent processes, followed by QTL analysis to determine whether genetic loci associated with the divergent dose responses contain genes identified by the co-expression analysis (see Figure 3 B for validation of divergent regulatory processes and Figure 4 B and C for validation of divergent molecular functions). This validation assumes that divergent co-expression relationships of small molecule targets leads to altered sensitivity to inhibition of the corresponding target pathways (Supplementary Figure 4). Validation of this type is limited to pathways whose inhibition by small molecules results in growth inhibition. In *P. falciparum*, for which conventional gene knockdowns are challenging, this approach provides a fast and

alternative and, furthermore, illustrates the general utility of co-expression networks for predicting vulnerable drug targets in a given phenotypic state (here CQR).

B. Establishing functional integrity of co-expression networks using synthetic lethal gene pairs

Synthetic lethal gene pairs functionally compensate for each other (Supplementary Figure 1 A). As a consequence, co-expression of these genes is highly constrained. The need for this constraint is supported by the evolutionary conservation of synthetic lethal interactions in different species [1], the conservation of their co-expression [2, 3], a propensity for negative correlations between their transcript levels [4] and upregulation of duplicated genes following deletion of their paralogs [5, 6]. Based on this, we assessed the integrity of the co-expression networks for CQR and CQS progeny by examining the co-expression relationships existing between previously reported synthetic lethal gene pairs [7]. As shown in Supplementary Figure 1 below, co-expression between synthetic lethal gene pairs tend to be negatively correlated in agreement with similar observations in synthetic lethal human genes [4].

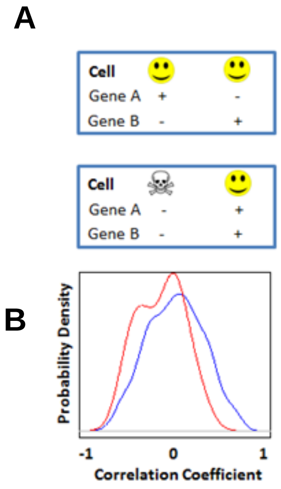

C

SyntheticLethalGeneA	SyntheticLethalGeneB	CQS	CQR	RewiringClass
PF3D7_1102400	PF3D7_0412300	0.03	-0.16	NoCorrelation
PF3D7_1120100	PF3D7_0413500	0.16	-0.06	NoCorrelation
PF3D7_1012500	PF3D7_0514600	-0.008	-0.52	GainOfNegative
PF3D7_0513300	PF3D7_0514600	-0.24	0.04	NoCorrelation
PF3D7_1008900	PF3D7_0415600	0.26	0.13	NoCorrelation
PF3D7_1456800	PF3D7_0316300.1	-0.62	-0.51	Stable
PF3D7_1340900	PF3D7_1206100	-0.08	-0.53	GainOfNegative
PF3D7_1366500	PF3D7_0605600	-0.65	-0.24	LossOfNegative
PF3D7_1315600	PF3D7_1410200	0.19	0.26	NoCorrelation
PF3D7_1315600	PF3D7_1340900	-0.09	-0.81	GainOfNegative
PF3D7_1409900	PF3D7_1410200	-0.05	0.11	NoCorrelation
PF3D7_1340900	PF3D7_1409900	0.23	-0.16	NoCorrelation
PF3D7_0316600	PF3D7_0915000	-0.06	0.02	NoCorrelation
PF3D7_1034400	PF3D7_0316600	-0.3	-0.35	NoCorrelation

Supplementary Figure 1. Co-expression of synthetic lethal pairs obtained from flux balance analysis [7]. (A) Schematic representation of synthetic lethal gene pairs (gene A and B). Deletion of either gene alone is not deleterious while simultaneous deletion of both genes leads to cell death. (B) Synthetic lethal pairs from *P. falciparum* are more likely to have negative correlation relationships (red line) compared to randomly sampled gene pairs (blue line, Wilcoxon test, *P* = 0.05). This is consistent with observations in other species [4]. (C) Table showing spearman correlation coefficients between synthetic lethal gene pairs. Highlighted in red is the conserved synthetic lethal co-expression relationship. Yellow are synthetic lethal co-expression relationships that have correlation coefficient greater than 0.5.

C. Triangular Inequality Prioritization of Interactions (TrIPI): Identifying functional partners

The presence of indirect correlations between genes confounds the use of gene correlations to infer functional relations. These drawbacks have been mitigated through the use of partial correlation analysis [8], triangle reduction [9], data processing inequality [10] and context likelihood of relatedness (CLR) in the case of networks

based on mutual information [11]. At the time of writing this paper, two additional methods for pruning indirect interactions using fundamental properties in the dynamical correlations in networks [12] and eigen-decomposition combined with infinite-sum series appeared [13]. The TrIPI approach takes advantage of the structure of correlation relationships and applies a simple heuristic based on the triangle inequality to prioritize the interactions into direct partners for a given gene and regulatory candidates of its co-expression network. The given gene of interest reduces the hypothesis search space normally encountered when doing genome-wide pairwise correlation analysis. Unlike all the other methods, TrIPI not only predicts direct interactions but also prioritizes indirect interactions that have regulatory implications.

Given a gene of interest Q, TrIPI constructs triangles whose sides are correlation coefficients between Q, and every other pair of genes whose correlation to Q have passed a baseline threshold (Figure 3). It then computes a statistic- the transitivity score – for each gene. The transitivity score, *t*, measures the number of triangles in which the magnitude of the correlation between Q and a second gene R is consistent with the triangle inequality. A score of zero implies that in the context of the correlation between Q and any other possible gene, the correlation between Q and R is always the highest. Therefore, Q and R are very tightly co-expressed and are referred to as direct partners. On the other hand, for the relationship QS (Supplementary Figure 3), *t* equals 1 since the triangular inequality $\min(|QR|, |RS|) > |QS|$ and $sign(QR.RS) = sign(QS)$ holds.

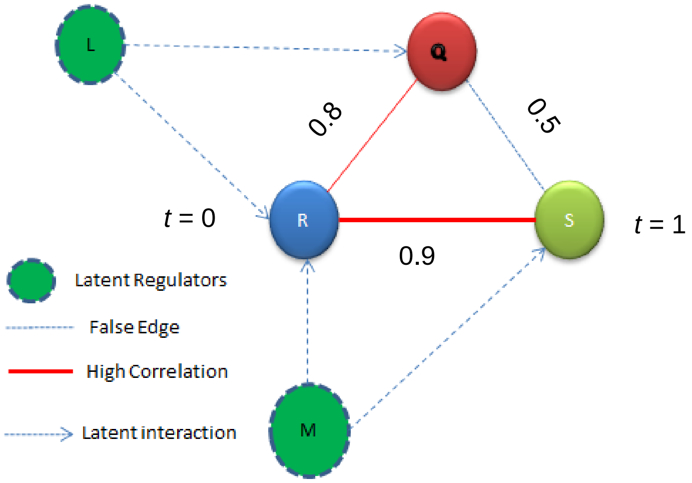

Supplementary Figure 2: Given 3 genes Q, R and S where both Q and R are regulated by unobserved regulator L while R and S are regulated by the latent regulator M, an indirect correlation arises between Q and S. If the correlation between Q and S is larger than that of QR and RS, then the edge QS cannot be sufficiently explained by indirect effects of L and M on S. In contrast, when QS is the smallest of the correlations in the triangle, then indirect effects of L and M on S may not be ruled out. This condition is a necessary but not sufficient condition for an indirect connection between Q and S [9]. A high *t* of say *n* implies that in the context of *n* third party nodes, QS is always the weaker of the correlations. Thus, a higher *t* provides corroborating evidence that QS is potentially a weaker connection. The mathematical basis supporting the ability of triangle reduction to prune indirect correlations has been described previously [9].

D. Application of TrIPI to infer regulatory relationships

While a low *t* implies direct interactions, genes with extremely high *t* have a unique topological position in the co-expression network anchored on a gene of interest. Such genes are connected to direct neighbors of Q hence can be viewed as local hubs.

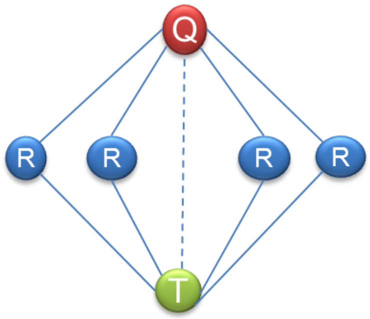

Supplementary Figure 3. Application of TrIPI for the prediction of regulatory candidates**.** Although node T appears indirectly connected to Q, its direct connection to all neighbors of node Q makes it an important gene in Q's co-expression network. In particular, T provides the shortest path to visit all neighbors of Q and is therefore an optimal position for influencing functions of Q that are mediated through its partners.

E. Prediction of diverging drug susceptibility from diverging co-expression networks

Here we describe the conceptual basis for using co-expression networks to predict compromised functional interactions which could result into altered drug response. Our main assumption is that the functional integrity of interacting genes requires their coordinated expression. This is evidenced by the high conservation of co-expression networks across species [1, 14, 15], the preponderance of negative correlations between transcripts levels encoded by synthetic lethal gene pairs [4] or paralogs [5, 6] and the ability of gene expression to mask the effects of mutations through epigenetic epistatic interactions [16, 17]. We hypothesize that divergence in co-expression relationships can result into new genetic dependencies and compromise or enhance response to secondary perturbations. For example, given two synthetic lethal gene pairs

(A and B, Supplementary Figure 4) whose co-expression is negatively correlated under wild-type conditions, exposure of the cells to a drug that inhibits only A would not result in death since B would become upregulated in response and compensate for inhibition of A. Perturbations that decouple the co-expression of A and B would abrogate this compensatory effect leading to sensitivity of the cells to inhibition of A. Thus, changes in co-expression relationships can lead to differential drug susceptibility.

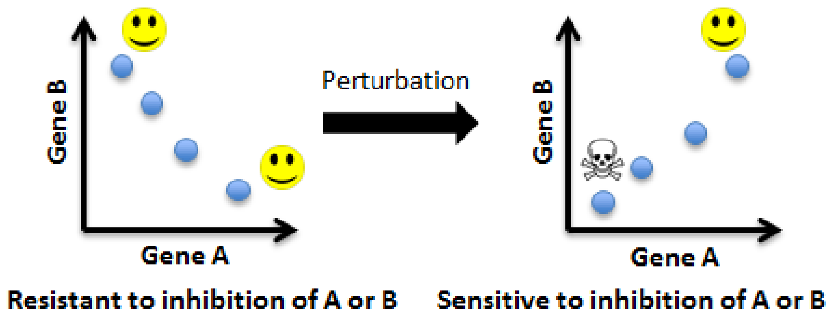

Supplementary Figure 4: Example of how genetic interactions can become compromised by changes in co-expression relationship. Gene A and B are synthetically lethal: on inhibition of A, compensation occurs through upregulation of B. This relationship is captured as negative correlations between transcript levels of A and B. This compensation becomes disrupted when the regulatory relationship between synthetic lethal gene pairs A and B is positive such that inhibition of A induces the regulatory system to downregulate B. A similar approach can be extended to co-expression changes involving other critical genetic interactions such as synthetically viable or positive genetic interactions.

F. Regulatory models for co-expression divergence

We consider two models for the observed differential co-expression of *pfcrt* in CQR and CQS parasites (Supplementary Figure 5).

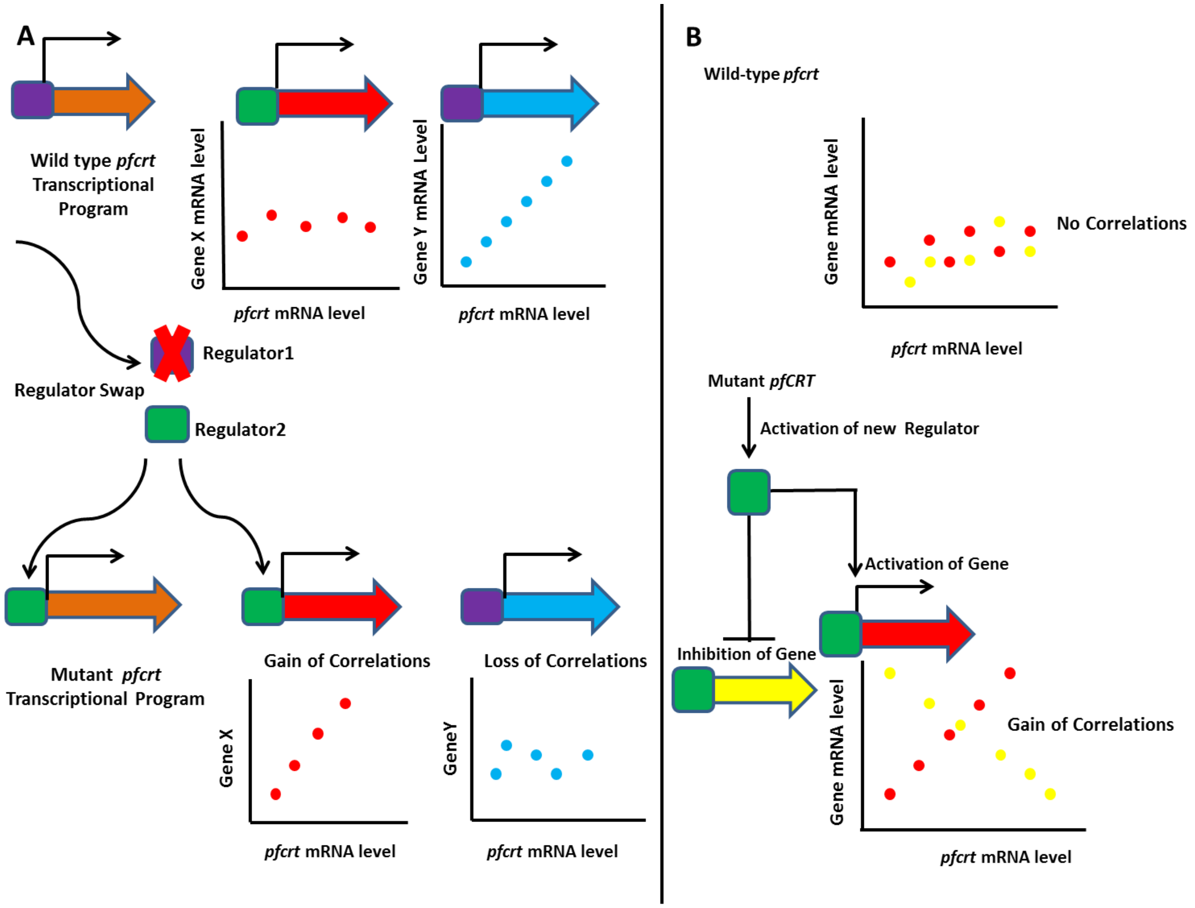

Supplementary Figure 5. Two proposed models for the regulatory divergence between CQR and CQS progeny clones. (A) A regulon handover model in which *pfcrt* is recruited into a new regulon in CQR parasites. In wild type parasites, both *pfcrt* and Gene Y (blue) are regulated by Regulator 1 leading to a positive correlation between the levels of the 2 genes. Gene X (red) and *pfcrt* are however regulated by different regulators and hence their mRNA levels are not correlated. In mutant parasites, a regulator swap occurs and *pfcrt* is now under control of Regulator 2 which also controls Gene X. As a

consequence, expression of *pfcrt* is now coupled to expression of Gene X (gain of positive correlation) and decoupled from Gene Y (loss of positive correlation). (B) A physiologically mediated divergence in which the mutant *pfcrt* gene codes for a transporter that is associated with altered transport of specific metabolites leading to activation of transcriptional programs that are not normally activated by *pfcrt.* Activation of a new regulator leads to activation of Gene X (red) and inactivation of Gene Z (yellow) leading to a gain of positive and negative correlation respectively.

References

1. Dixon SJ, Fedyshyn Y, Koh JL, Prasad TS, Chahwan C, Chua G, Toufighi K, Baryshnikova A, Hayles J, Hoe KL, Kim DU, Park HO, Myers CL, Pandey A, Durocher D, Andrews BJ, Boone C: **Significant conservation of synthetic lethal genetic interaction networks between distantly related eukaryotes.** Proc Natl Acad Sci U S A 2008, **105**(43):16653-16658.

2. Koch EN, Costanzo M, Bellay J, Deshpande R, Chatfield-Reed K, Chua G, D'Urso G, Andrews BJ, Boone C, Myers CL: **Conserved rules govern genetic interaction degree across species.** Genome Biol 2012, **13**(7):R57.

3. Nijman SM: **Synthetic lethality: general principles, utility and detection using genetic screens in human cells.** FEBS Lett 2011, **585**(1):1-6.

4. Folger O, Jerby L, Frezza C, Gottlieb E, Ruppin E, Shlomi T: **Predicting selective drug targets in cancer through metabolic networks.** Mol Syst Biol 2011, **7**:501.

5. DeLuna A, Springer M, Kirschner MW, Kishony R: **Need-based up-regulation of protein levels in response to deletion of their duplicate genes.** PLoS Biol 2010, **8**(3):e1000347.

6. DeLuna A, Vetsigian K, Shoresh N, Hegreness M, Colon-Gonzalez M, Chao S, Kishony R: **Exposing the fitness contribution of duplicated genes.** Nat Genet 2008, **40**(5):676-681.

7. Plata G, Hsiao TL, Olszewski KL, Llinas M, Vitkup D: **Reconstruction and flux-balance analysis of the Plasmodium falciparum metabolic network.** Mol Syst Biol 2010, **6**:408.

8. Johansson A, Loset M, Mundal SB, Johnson MP, Freed KA, Fenstad MH, Moses EK, Austgulen R, Blangero J: **Partial correlation network analyses to detect altered gene interactions in human disease: using preeclampsia as a model.** Hum Genet 2011, **129**(1):25-34.

9. Rice JJ, Tu Y, Stolovitzky G: **Reconstructing biological networks using conditional correlation analysis.** Bioinformatics 2005, **21**(6):765-773.

10. Basso K, Margolin AA, Stolovitzky G, Klein U, Dalla-Favera R, Califano A: **Reverse engineering of regulatory networks in human B cells.** Nat Genet 2005, **37**(4):382-390.

11. Faith JJ, Hayete B, Thaden JT, Mogno I, Wierzbowski J, Cottarel G, Kasif S, Collins JJ, Gardner TS: **Large-scale mapping and validation of Escherichia coli transcriptional regulation from a compendium of expression profiles.** PLoS Biol 2007, **5**(1):e8.

12. Barzel B, Barabasi AL: **Network link prediction by global silencing of indirect correlations.** Nat Biotechnol 2013, **31**(8):720-725.

13. Feizi S, Marbach D, Medard M, Kellis M: **Network deconvolution as a general method to distinguish direct dependencies in networks.** Nat Biotechnol 2013, **31**(8):726-733.

14. Oldham MC, Horvath S, Geschwind DH: **Conservation and evolution of gene coexpression networks in human and chimpanzee brains.** Proc Natl Acad Sci U S A 2006, **103**(47):17973-17978.

15. Jordan IK, Marino-Ramirez L, Wolf YI, Koonin EV: **Conservation and coevolution in the scale-free human gene coexpression network.** Mol Biol Evol 2004, **21**(11):2058-2070.

16. Park S, Lehner B: **Epigenetic epistatic interactions constrain the evolution of gene expression.** Mol Syst Biol 2013, **9**:645.

17. Burga A, Casanueva MO, Lehner B: **Predicting mutation outcome from early stochastic variation in genetic interaction partners.** Nature 2011, **480**(7376):250-253.
